# Gender-based violence response and health system readiness during emergencies in Sierra Leone: a qualitative study of survivor experience, facility preparedness, and referral pathways

**DOI:** 10.1080/16549716.2026.2740361

**Published:** 2026-09-29

**Authors:** Augustus Osborne, Ahmed Vandy, Camilla Bangura

**Affiliations:** a Institute for Development, Freetown, Sierra Leone; b College of Medicine and Allied Health Sciences, University of Sierra Leone, Freetown, Sierra Leone; c Department of Biological Sciences, School of Basic Sciences, Njala University, Freetown, Sierra Leone

**Keywords:** Humanitarian preparedness, emergency contraception, post-exposure prophylaxis, psychosocial support, health system resilience

## Abstract

**Background:**

Gender-based violence (GBV) intensifies during emergencies, yet health and protection systems in Sierra Leone remain inadequately prepared to respond. Survivors face barriers including stigma, breaches of confidentiality, and weak referral pathways.

**Objective:**

This study explored how health and protection systems in three districts of Sierra Leone respond to GBV during emergencies.

**Methods:**

A qualitative descriptive study was conducted across three districts, integrating semi-structured interviews with referral pathway mapping. Purposive sampling recruited 54 participants: 48 key informants across eight stakeholder groups and 6 survivors identified through referral organisations. Data were analysed using the framework method. Data collection took place outside an active emergency; emergency-specific findings therefore reflect participant recollection rather than direct observation.

**Results:**

Four themes emerged: fragmented and under-resourced clinical readiness; confidentiality failures and stigma as structural barriers; dysfunctional referral pathways and coordination gaps; and limited, inconsistent survivor-centred psychosocial care. Participants reported that emergency contraception was frequently unavailable and, in some accounts, withheld on moral grounds. Referrals to police and social welfare were described as ad hoc, reliant on personal relationships, and severely disrupted when GBV was excluded from emergency coordination structures. Counsellors were largely absent from peripheral facilities. A minority of participants reported functioning one-stop centre care.

**Conclusions:**

Participants described GBV response arrangements that were fragile in stable periods and, in their recollection of previous emergencies, substantially weakened, alongside a smaller set of components that functioned. Emergency preparedness planning would benefit from explicit attention to survivor-centred GBV care; the applicability of these district-level findings elsewhere requires further study.

## Paper Context

- **Main findings:** In three districts of Sierra Leone, survivors and response actors described fragmented clinical care, breaches of confidentiality, referral pathways that were severely disrupted during previous emergencies, and limited survivor-centred psychosocial support, alongside a small number of components, principally the one-stop centre model, which were described as working as intended.
- **Added knowledge:** The study provides fine-grained evidence of how survivor experiences, provider attitudes, and community norms intersect to undermine effective gender-based violence response, highlighting the reported disruption of referral systems during previous emergencies and the moral gatekeeping of emergency contraception alongside stock shortages.
- **Global health impact for policy and action:** Findings indicate that emergency preparedness frameworks would benefit from explicitly embedding survivor-centred care, positioning gender-based violence response as a core component of health system resilience and of global humanitarian policy rather than as a post-crisis addition, and they identify existing one-stop centre and police family support structures as the most feasible entry points for that work.

## Background

Gender-based violence (GBV) is among the most pervasive yet systematically neglected public health and human rights crises of our time. One in three women worldwide will experience physical or sexual violence in their lifetime, and prevalence rises sharply in humanitarian and emergency settings [1,2]. Emergencies, whether driven by disease outbreaks, floods, conflict, or political instability, reliably amplify GBV. Systematic review evidence confirms that rates of sexual violence rise substantially in conflict-affected and displaced populations [3]. Displacement removes the private space needed for disclosure; the breakdown of community protection eliminates the pathways through which survivors reach services; and the collapse of formal services erodes the responses on which help-seeking depends [4]. As a result, GBV becomes most prevalent precisely when systems are least able to respond. In this study, emergency refers to an acute, population-level shock that disrupts routine health and protection services, and participants were asked about the 2014 to 2016 Ebola virus disease outbreak, the 2017 Regent landslide, the COVID-19 pandemic, and the floods of 2022 and 2023.

Sierra Leone carries one of the highest documented rates of GBV in West Africa; a 2019 national survey estimated that more than 6 in 10 women aged 15–49 had experienced physical or sexual violence [5]. This burden sits within a post-conflict society, a health system weakened by Ebola, and communities in which patriarchal norms, early and forced marriage, female genital mutilation, and domestic violence remain normalised [6]. During Ebola, health workers were redeployed, referral systems ceased to operate, and community protection networks were severed by quarantine [7], and women who experienced violence during the Ebola and COVID-19 outbreaks faced compounded barriers to help-seeking, including fear that presenting at a facility would result in quarantine [8].

The policy architecture has advanced since the Sexual Offences Act and the Domestic Violence Act of 2012 [9]. The national GBV protocol, updated in 2018, mandates survivor-centred care across health, justice, and social welfare, and the Rainbo Initiative operates one-stop centres providing integrated clinical and psychosocial services in several urban districts [10]. The Family Support Units (FSUs) of the Sierra Leone Police are the formal mechanism for survivor referral and investigation. Both are considered later entry points for strengthening. On paper, the architecture is coherent; in practice, the gap between policy and implementation is wide, and it widens when emergencies strike.

Readiness for GBV response during emergencies requires trained staff, private consultation space, uninterrupted supplies of post-exposure prophylaxis (PEP) and emergency contraception (EC), functioning referral to police, social welfare, legal aid, and psychosocial services, and an institutional culture that places the survivor’s safety, dignity, and autonomy at the centre of every interaction [11]. The World Health Organization (WHO) Health Emergency and Disaster Risk Management (Health EDRM) framework identifies GBV response as a core component of health system resilience [12], yet these requirements are rarely met in Sierra Leone and comparable settings even outside emergencies [13]. The social-ecological model explains why: stigma, family pressure to settle privately, norms of male authority, and under-resourced facilities and fragmented coordination together produce a response that is nominally available but functionally inaccessible, and emergencies intensify every level at once [14]. The rights-based approach frames GBV care as a justiciable entitlement [15], not a donor-funded addition to be disrupted first when funding contracts during emergencies [16].

Semi-structured interviews and referral pathway mapping trace the survivor’s journey from disclosure to care and identify where and why systems fail [17]. The research question guiding this study is as follows: how prepared are health and protection systems in three districts of Sierra Leone to respond to GBV during emergencies? Its objectives are to explore the barriers survivors face in accessing timely care and referral; to explore facility readiness to provide survivor-centred services; to analyse coordination between health, police, social welfare, non-governmental organisations (NGOs), and community actors; and to develop evidence-informed recommendations to strengthen GBV response during emergencies.

## Methods

### Study design

This qualitative descriptive study integrated semi-structured interviews with referral pathway mapping and was analysed using the framework method [18,19]. A descriptive approach was chosen to produce a low-inference account of how the GBV response is organised, delivered, and experienced, usable by policy and programme actors [20], and mapping allowed comparison between documented referral protocols and practice. The study is reported in accordance with the Consolidated Criteria for Reporting Qualitative Research (COREQ) checklist (Additional file 1) [21]. Data were collected between January and June 2024, when no major emergency was active in any study district; the study therefore documents readiness as reported at that time together with retrospective accounts of previous emergencies, distinguished throughout, and makes no claim to direct observation of system performance during an emergency. The conceptual framework is summarised in Figure 1.

**Figure 1.**
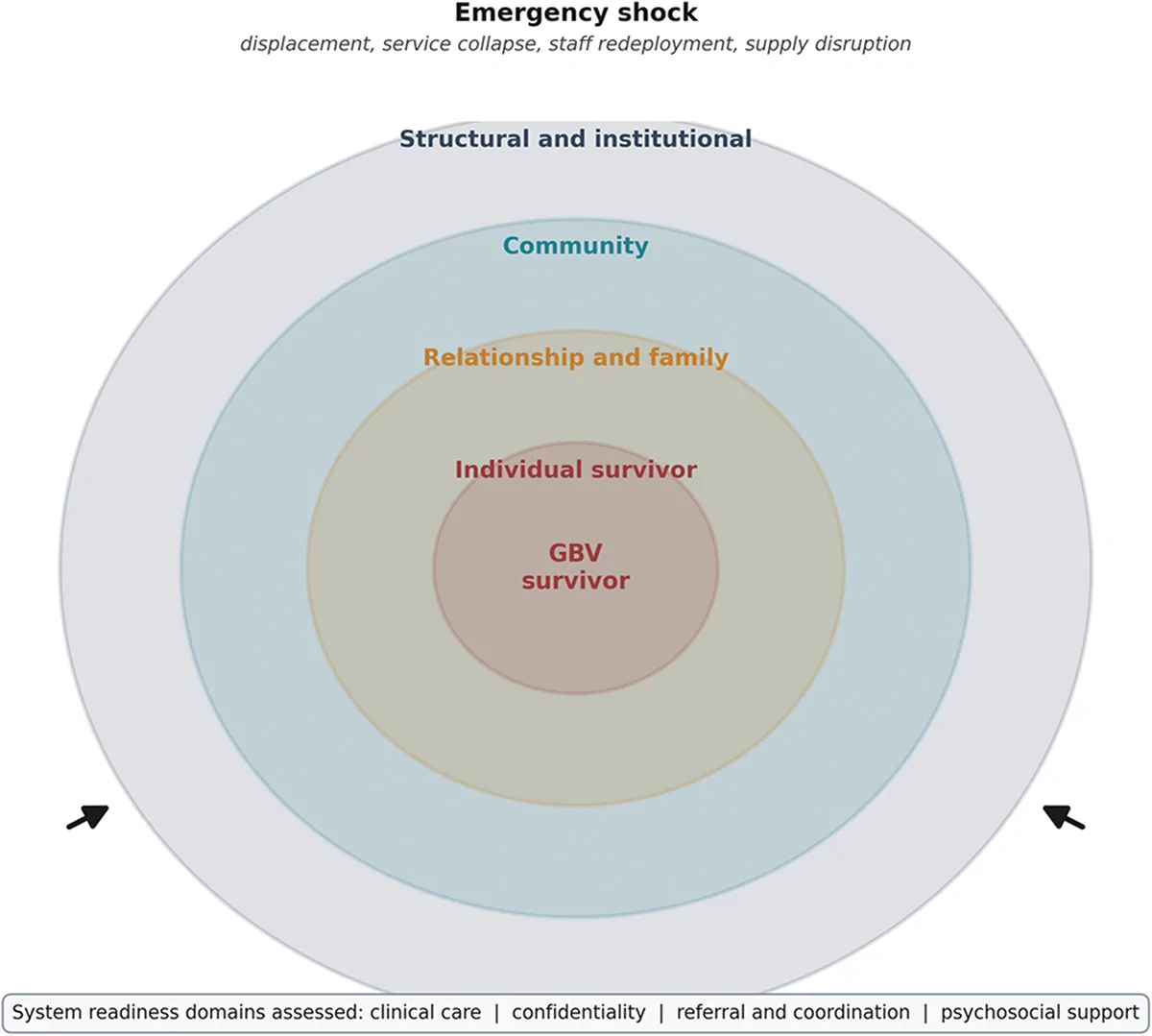
Conceptual framework: the social-ecological model of GBV response readiness under emergency stress, adapted by the authors from the social-ecological model [14] and the health EDRM framework [12].

### Study setting

Data were collected in Western Area Urban (Freetown), Kenema District, and Port Loko District, selected for variation in service infrastructure, emergency history, and geography: Freetown is the most densely serviced setting, with one-stop centres, referral hospitals, and NGO protection actors; Kenema has experienced conflict-era displacement and Ebola response; and Port Loko, predominantly rural, represents peripheral conditions where preparedness and GBV service capacity are most limited. These are 3 of the 16 districts of Sierra Leone, and the findings describe the GBV response in these districts rather than nationally.

### Participants and sampling strategy

Participants were purposively sampled from a frame constructed with district health management teams, the police FSUs, and NGO protection coordinators. Forty-eight key informants were recruited across eight stakeholder groups: healthcare providers (*n* = 11); social workers and social welfare officers (*n* = 6); police FSU personnel (*n* = 6); NGO protection officers and GBV programme staff (*n* = 7); community and religious leaders (*n* = 5); women’s group representatives and community health volunteers (*n* = 3); psychosocial counsellors (*n* = 5); and district emergency response coordinators (*n* = 5). In addition, survivors of GBV (*n* = 6) were included, giving a total of 54 participants (Table 1; Figure 2). Key informants were eligible if aged 18 years or older, had held a role with defined GBV responsibilities in a study district for at least 2 years, and had handled at least one GBV case in the preceding 12 months.

**Figure 2.**
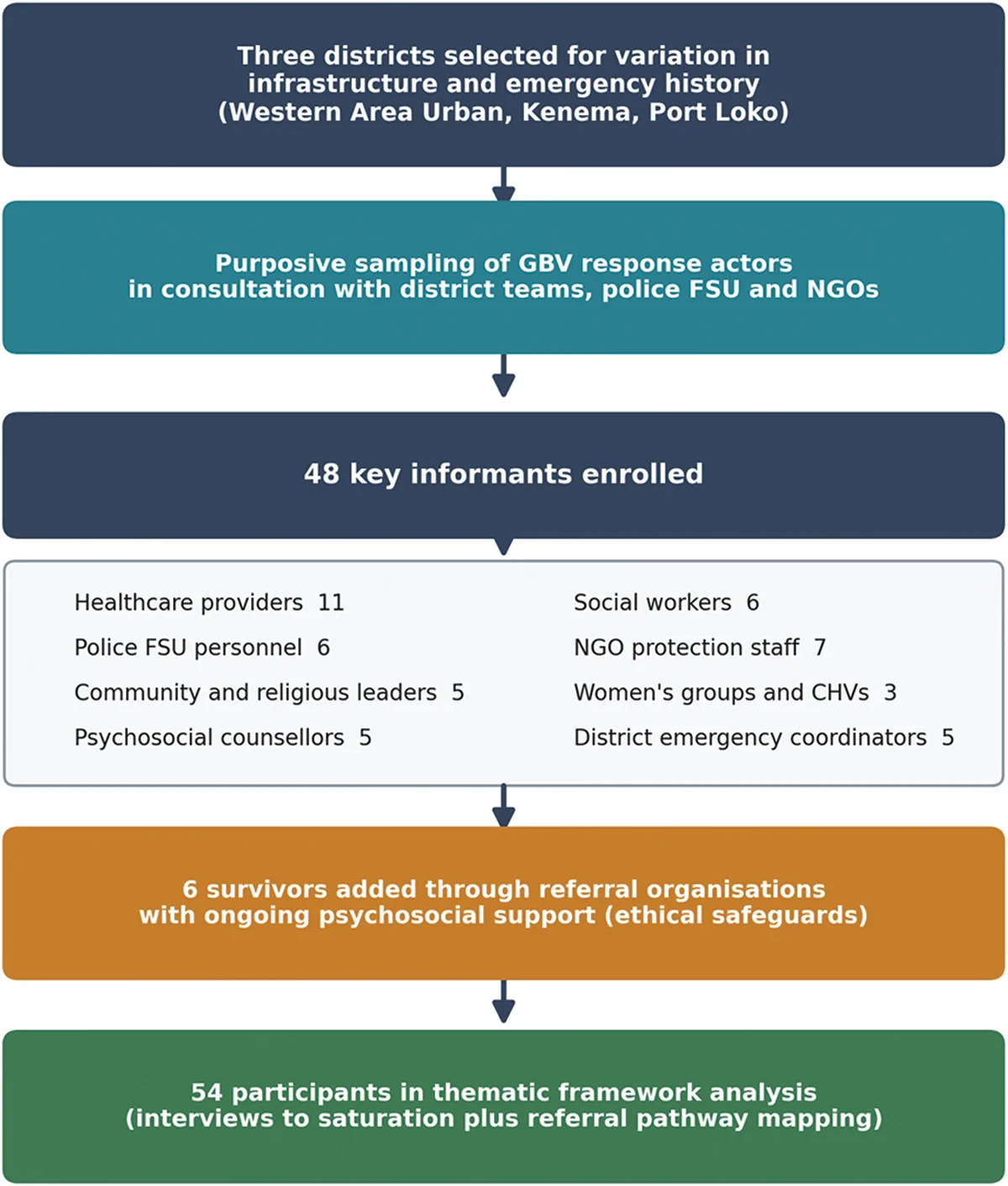
Study profile: district selection, purposive sampling of 48 key informants, addition of six survivors through referral organisations, and the final sample of 54.

**Table 1.** Characteristics of study participants (N = 54).

Characteristic	n
**Key informants (stakeholder groups)**	**48**
Healthcare providers (nurses, midwives, medical officers)	11
Social workers and social welfare officers	6
Police FSU personnel	6
NGO protection and programme staff	7
Community and religious leaders	5
Women’s group representatives and community health volunteers	3
Psychosocial counsellors	5
District emergency response coordinators	5
**Survivors (recruited through referral organisations)**	**6**
**Sex**	
Women	37
Men	17
**Age range (years)**	**19 to 58**
**District distribution of key informants**	
Western Area Urban	19
Kenema	16
Port Loko	13

Survivors were never approached by the research team in the first instance. The Rainbo Initiative and a partnering NGO, already providing them with psychosocial support, identified clients aged 18 years or older, at least 6 months from the index event, not in acute crisis or ongoing danger, and judged by their counsellor as clinically stable; the counsellor made the initial approach, and only those who then contacted the team were enrolled. Survivor transcripts were coded within the same framework as key informant transcripts so that survivor and provider accounts of the same points in the pathway could be read against each other and were used to test provider accounts and validate the referral maps, not to estimate the frequency of any experience. Recruitment continued until saturation, assessed prospectively for each stakeholder group using the code-based approach of Hennink and Kaiser [22]; it was reached before the final interview in every key informant group and was neither sought nor claimed for survivors (Supplementary file 3).

Survivors were all women aged 19–58 years (four had experienced sexual violence and two had severe domestic violence), recruited across the three districts through referral organisations, and all were receiving ongoing psychosocial support.

### Ethical considerations and GBV-specific safeguards

Ethics approval was granted by the Sierra Leone Ethics and Scientific Review Committee (SLESRC/2023/GBV/047), with the survivor component approved as a separate protocol with its own information sheet, consent form, and distress protocol, and the study complied with the Declaration of Helsinki and the WHO ethical and safety recommendations for research on violence against women [23]. All participants gave written informed consent, with witnessed oral consent for those with limited literacy. Survivor interviews were conducted by trained female researchers with GBV counselling backgrounds, with a named counsellor available on the day and defined stopping rules, and no identifying details about the index event or perpetrator were collected.

### Data collection procedures

Semi-structured interview guides were developed from the study objectives, the survivor-centred care framework, the Health EDRM framework, the social-ecological model, and the rights-based approach to health [12,14,15,24]. Interview guides were designed to explore five domains: clinical readiness and commodity availability; confidentiality, privacy, and provider conduct; referral and inter-agency coordination; psychosocial support and survivor-centred practice; and emergency-specific experience and preparedness. The full guides, comprising the core guide, the group-specific modules, the survivor guide, and a table mapping each guiding framework to the corresponding guide items, are provided in Supplementary file 1, and the operationalisation of the frameworks is described in Supplementary file 3. A single core guide with role-specific modules was used with all key informant groups, and a separate narrative journey guide, with no fixed question order and no questions about the index event, with survivors. Guides were piloted with two healthcare providers and two NGO staff. Interviews in English, Krio, or Mende lasted 30–60 minutes and were audio-recorded and transcribed verbatim in the language of interview; translations into English were reviewed against the recording by a second bilingual team member, 20% were back-translated by a third, and none of the 34 discrepancies logged altered a code (Supplementary file 3).

### Referral pathway mapping

Mapping followed three steps. A documented pathway was reconstructed for each district from the 2018 national referral protocol, district coordination minutes, facility standard operating procedures, FSU case handling instructions, and referral forms in use. A functional pathway was then elicited in 22 mapping discussions (9 in Western Area Urban, 7 in Kenema, 6 in Port Loko) in which healthcare providers, social welfare officers, FSU officers, and NGO protection officers traced, step by step, the last three GBV cases they had handled, and then a case handled during a previous emergency. Each discussion produced a node and link diagram coded on whether each step was specified in protocol, routinely completed, and where it failed; diagrams were transcribed independently by two researchers, reconciled, compiled into district matrices for stable and emergency conditions, and compared to produce Figure 3, in which a link is functional only where both the referring and receiving actor group reported it as routinely completed. Draft maps were validated with 11 participants and against survivor accounts. The mapping was not audited against routine service records, and the emergency maps are participants’ reconstruction of past practice (Supplementary files 2 and 3).

**Figure 3.**
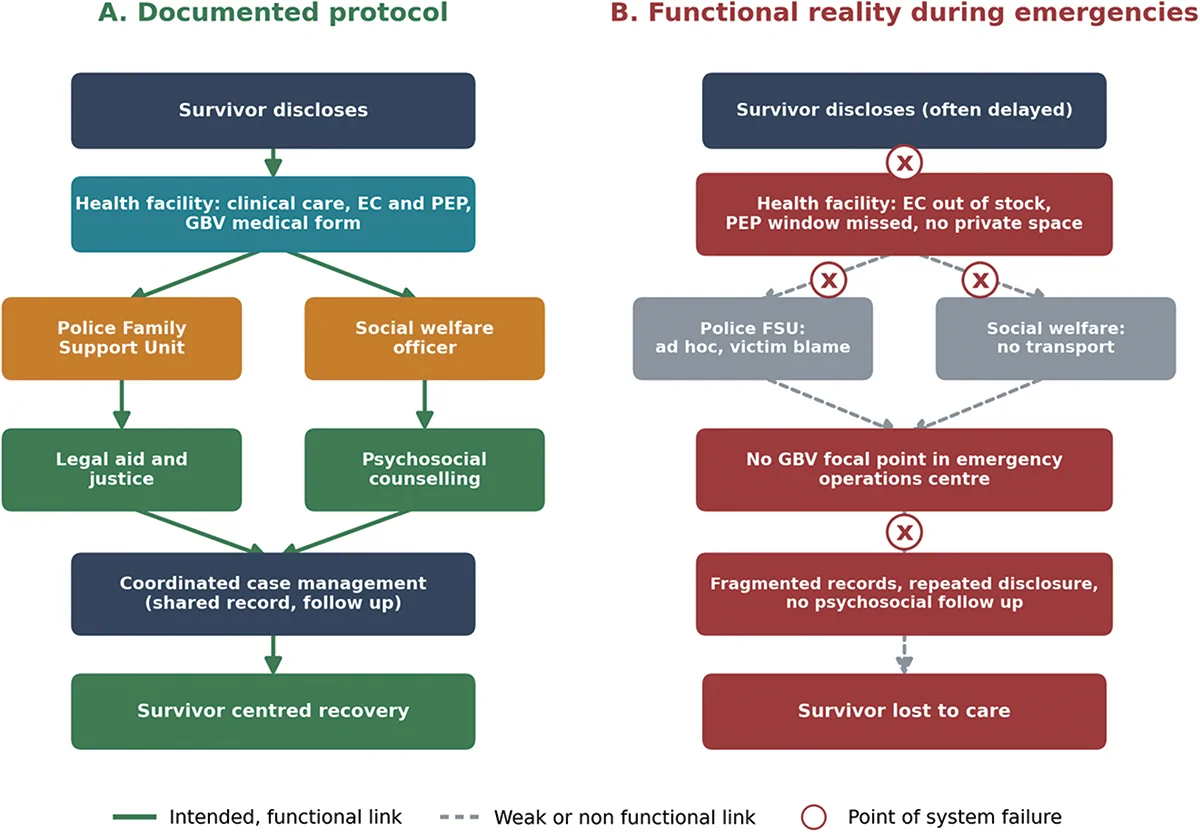
The GBV survivor referral pathway in the study districts: panel A, the documented protocol; panel B, the functional reality participants described during emergencies.

### Data analysis

The framework method was applied in five stages [18,19]: familiarisation with five transcripts spanning groups and districts; open coding of those transcripts, generating 214 initial codes grouped into a working framework that combined inductive codes with deductive codes from the guiding frameworks; indexing of all 54 transcripts, with the codebook revised twice, to a final framework of 4 themes, 11 subthemes, and 96 codes; charting into a framework matrix, one row per participant and one column per subtheme; and interpretation by column, by row, and across groups and districts to identify convergence, divergence, and deviant cases. NVivo 14 software was used to manage, code, and organize data. Two researchers coded independently, 20% of transcripts were double coded, and the 17 disagreements were resolved by consensus. Negative case analysis re-interrogated each subtheme for accounts that qualified or contradicted it, as reported in a dedicated Results subsection (Supplementary file 3).

### Trustworthiness and reflexivity

Trustworthiness was addressed against credibility, transferability, dependability, and confirmability [18] through method and source triangulation, cross-referencing of provider and survivor accounts, purposive selection of urban, mixed, and rural districts, independent double coding, an audit trail, reflexive memos, and negative case analysis (Supplementary file 3). The team included Sierra Leonean and international researchers in GBV programming, emergency health, public health, and clinical medicine, who reflected throughout on how their positions might shape interpretation.

## Results

Purposive sampling yielded 48 key informants across eight stakeholder groups and six survivors recruited through referral organisations, for a final sample of 54 (Figure 2).

### Overview of themes

Of the 54 participants, 37 (69%) were women and 17 (31%) were men, aged 19–58 years. Four themes emerged, shown with their subthemes in Figure 4, and a fifth subsection reports the divergent accounts, deviant cases, and functioning components identified through negative case analysis; participants are identified by role and district only. Accounts of current readiness describe conditions reported between January and June 2024, when no major emergency was active; accounts of emergency conditions are retrospective and relate to Ebola, the Regent landslide, COVID-19, and the floods of 2022 and 2023. Emergency-related findings are therefore reported as participant experiences and recollections and are attributed to participants throughout, rather than as directly observed system performance. Findings describe the three study districts, not the GBV response nationally, and additional quotations for every theme are provided in Supplementary file 4.

**Figure 4.**
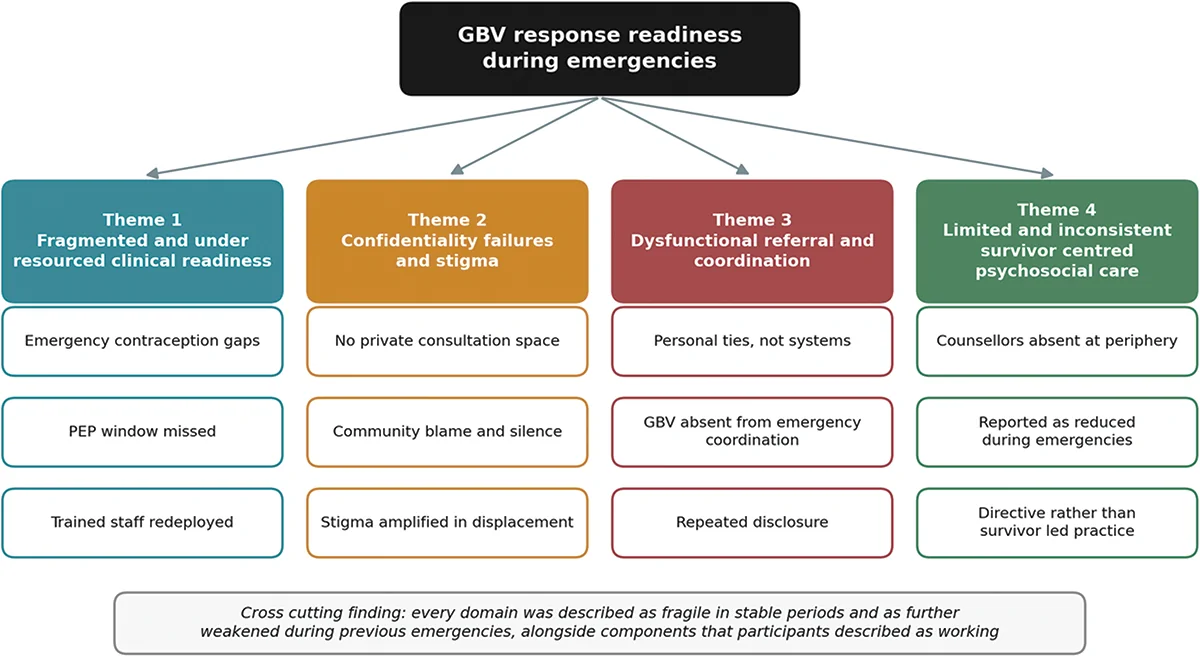
Thematic framework: four themes and their principal subthemes, each described as fragile in stable periods and further weakened during emergencies.

### Theme 1: fragmented and under-resourced clinical readiness

Clinical readiness was consistently described as inadequate, particularly in peripheral facilities, and participants recalled that during previous emergencies supply chains had been disrupted and staff redeployed, compounding the shortfalls described in stable conditions.

#### Emergency contraception availability and access

Emergency contraception was among the most frequently cited gaps, raised without prompting by 9 of the 11 healthcare providers, all 5 psychosocial counsellors, and 4 of the 6 survivors. Across the districts EC was reported as unavailable, inconsistently stocked, or withheld on moral or procedural grounds, but the distribution differed: participants in Port Loko located the problem almost entirely in stock, whereas provider refusal was described only in Western Area Urban and Kenema, where stock was more reliable. A nurse in Port Loko recalled the 2023 floods:

When the floods came, the supply chain broke down completely. We had no emergency contraception for almost three weeks. Women were coming to us after rape, and we had nothing to give them. We told them to go to the district hospital, but the roads were flooded. They could not go. (Nurse, Port Loko)

In Freetown, a medical officer at a referral hospital described colleagues who refused to give EC on grounds of belief, with no policy to override them, so that a woman who had been raped was sent away or told to return the next day (Supplementary file 4).

PEP for HIV was similarly affected. Providers in Kenema described a 72-hour window routinely missed because survivors arrived late, were turned away from the first facility they attended, or had not been told that PEP existed (Supplementary file 4).

#### Clinical examination and documentation

Forensic examination capacity was severely limited outside Freetown: in Port Loko and Kenema only one or two clinicians per district had been trained in clinical management of rape, none recently, and completion of the GBV medical form required for legal proceedings was described as inconsistent and poorly understood. Participants in all three districts recalled that during previous emergencies those trained clinicians were assigned to emergency duties, leaving facilities with untrained staff who sent survivors away (Supplementary file 4).

### Theme 2: confidentiality failures and stigma as structural barriers

#### Physical and procedural confidentiality failures

Confidentiality was described as persistently violated across all sites, as a systemic feature of the response rather than as isolated incidents. Dedicated private consultation space did not exist in the facilities represented, the space that did exist had been repurposed during previous emergencies, and survivors were examined in shared wards, consulted in corridors, or interviewed with doors open. A survivor described a peripheral health unit:

When I came to the clinic, there were other people waiting. The nurse called my name and asked me in front of everyone what happened. I could not say. I just said I fell. I was too ashamed to say the real thing in front of all those people. (Survivor, Port Loko)

Healthcare providers described the same conditions from their own vantage point, locating the difficulty in the physical infrastructure available to them (Supplementary file 4).

#### Community stigma and its interaction with help seeking

Community stigma was a powerful deterrent to disclosure. Across the districts, survivors, particularly of sexual violence, were described as blamed, ostracised by families and neighbours, and pressured to resolve violence through informal community mechanisms. Community leaders described positions that combined more than one consideration. Several spoke about both their concern for survivors and their expectations regarding disclosure, reputation, and marriage prospects:

We care about our women. But if a woman goes to the police and the story comes out, her reputation is finished. No man will marry her. Her family will suffer. Sometimes it is better to settle quietly. We are not saying what happened is right, but we are thinking about her future. (Community leader, Kenema)

Women’s group representatives described the pressure to settle as a mechanism that protected perpetrators and silenced survivors: the chief calls a meeting, the man pays a fine, and the woman, who has no voice in that meeting, is told to forgive and move on (Supplementary file 4).

Participants recalled that stigma intensified in displacement: survivors in temporary shelters described heightened surveillance, loss of social networks, and confinement with perpetrators, and said women did not report because the whole camp would know by morning (Supplementary file 4).

### Theme 3: dysfunctional referral pathways and inter-agency coordination gaps

Mapping showed a wide divergence between documented protocols and reported practice (Figure 3): formal pathways existed on paper in all three districts, but the communication systems, transport, inter-agency agreements, and trained personnel required to operate them were reported as absent or non-functional in most settings. Interviews and referral pathway mapping suggested that referral pathways were severely disrupted during previous emergency events.

#### Health to police and social welfare referrals

Referrals from health facilities to FSUs were described as inconsistent, delayed, and dependent on the personal initiative of individual staff, and police family support personnel expressed frustration with receiving incomplete referrals and at the absence of standardised information sharing procedures, describing a system of personal relationships in which the survivor had often left before the FSU arrived (Supplementary file 4).

Social welfare officers described similar fragmentation compounded by their own constraints; one officer covering three chiefdoms had no vehicle, phone credit, or transport budget, and by the time a family was reached the survivor had often been pressured to withdraw (Supplementary file 4). Participants recalled that during previous emergencies these pre-existing weaknesses became acute: communication failed, roads were impassable, and coordination meetings were suspended:

In an emergency, everyone is focused on the immediate crisis. Gender based violence is not on the emergency coordination agenda. There is no gender-based violence focal point in the emergency operations centre. The referral pathway that exists on paper simply does not function. (District emergency coordinator, Kenema)

#### NGO and community actor coordination

NGO protection officers described coordination that was fragmented in stable periods and dysfunctional during emergencies, with each organisation holding its own database and referral form and no unified case management system, so that a survivor had to tell her story three or four times to different people (Supplementary file 4).

Community health volunteers, often the first point of contact in rural areas, reported no training in GBV referral and no guidance on where to direct survivors and described themselves as helping in the dark (Supplementary file 4).

### Theme 4: limited and inconsistent survivor-centred psychosocial care, reported as further reduced during emergencies

Psychosocial support was identified as the most under-resourced component of the response. Counsellors were reported at one-stop centres and district hospitals but not at community health centres or health posts, so availability depended on where a survivor first presented, and participants recalled that during previous emergencies even these limited counselling resources were disrupted (Supplementary file 4). Survivors described the absence of support as a profound gap:

After what happened to me, I needed someone to talk to. The nurse was kind, but she was busy. She gave me medicine and told me to come back in two weeks. I went home alone. I had no one to talk to. I could not sleep for months. (Survivor, Western Area Urban)

#### Survivor-centred care in practice

The survivor-centred care framework, although referenced in national protocols, was poorly understood and inconsistently applied. Several healthcare providers described directing survivors towards particular courses of action, such as reporting, medication, or shelter, because they believed they knew what was best, and NGO officers, who articulated the principles more consistently, acknowledged that emergency pressure led them to move a survivor to safety without asking what she wanted (Supplementary file 4).

### Divergent accounts, deviant cases, and components that functioned

Negative case analysis identified accounts that qualified or contradicted each theme; they are summarised here and reported in full in Supplementary file 4. The clearest counter case was the Rainbo Initiative one-stop centres, which every actor category in Western Area Urban and Kenema described as the part of the system that worked as intended, attributing this to co-location of clinical care, counselling, and a police desk in one confidential building, a standing case file shared across those functions, and a dedicated commodity stock held outside the general pharmacy. Two of the six survivors had reached a one-stop centre:

At that place they took me into a room and closed the door. One person asked me the questions, not everybody. The medicine was there that same day. The police person came to me, I did not have to go to the station. I told my story one time. (Survivor, Western Area Urban)

In Western Area Urban, FSU officers and hospital staff independently described an informal messaging group linking the FSU duty officer, two clinicians, and an NGO caseworker, which had sustained referrals through the communication disruptions of the COVID-19 period and which they described as effective but unwritten and dependent on the individuals in it (Supplementary file 4).

Community leadership was similarly divided: section chiefs in Kenema and Port Loko were described who required suspected cases to be taken to the FSU, and one chiefdom bye-law made concealment of a sexual offence sanctionable. Provider refusal of EC was not described in Port Loko, so the two barriers to EC are not uniformly distributed, and three providers kept a personal reserve of EC and PEP for stockouts. Survivor accounts were not uniformly negative: two of six described respectful clinical care, four named an individual who had helped them, and criticism was directed more at the organisation of services than at individuals.

### Synthesis of readiness gaps across study districts

Table 2 synthesises the key GBV response domains assessed in this study, comparing documented protocol requirements with practice as reported by participants and identifying critical readiness gaps across the three study districts.

**Table 2.** GBV response domains, protocol requirements, observed practice, and critical readiness gaps.

Response domain	Protocol requirement	Reported practice	Critical readiness gap
EC	Available within 72 hours at all health facilities	Frequently out of stock; withheld by some providers on moral grounds	Supply chain failures during emergencies; no override policy for provider refusal
PEP	Initiated within 72 hours of exposure	Routinely missed due to late presentation and lack of survivor awareness	No community awareness of the window; referral delays exceed 72 hours
Clinical examination and documentation	Trained clinician; completed medical form	Undertrained staff; incomplete forms; trained staff redeployed during emergencies	Insufficient trained clinicians; no emergency staffing protocol
Confidential consultation space	Private room for all consultations	Absent in most peripheral facilities; shared spaces used routinely	No minimum infrastructure standard enforced; emergency repurposing of spaces
Psychosocial support	Counsellor available at point of care	Available only at district hospitals and one stop centres; absent during emergencies	No counsellors at peripheral facilities; no emergency psychosocial surge capacity
Police referral	Systematic referral with standardised information sharing	Ad hoc, dependent on personal relationships; delayed or absent during emergencies	No unified referral form; no communication protocol during emergencies
Social welfare referral	Timely social welfare officer response	Delayed by resource constraints; no transport or communication budget	Severely under resourced cadre; no emergency response role defined
Inter-agency coordination	Coordinated case management across sectors	Fragmented; no unified case management system; absent from emergency coordination	No focal point in emergency operations centres; no shared database
Community engagement	Community leaders as response partners	Ambivalent; some leaders reinforce informal settlement and stigma	No structured sensitisation programme linked to emergency preparedness
Survivor centred care	Survivor autonomy, informed consent, non-judgmental care	Inconsistently applied; paternalistic approaches common; emergency pressure reduces agency	No routine survivor centred care training; no emergency specific care protocol

The FSU is the police unit responsible for survivor referral and investigation.

## Discussion

In three Sierra Leonean districts, participants described GBV response arrangements that were fragile in stable conditions and, in their recollection of previous emergencies, further weakened, across clinical readiness, confidentiality and stigma, referral and coordination, and survivor-centred psychosocial care. Together these describe a pattern in which survivors become least visible to the system when they are most vulnerable, alongside a smaller set of components that participants described as working.

In pursuing these objectives, this study contributes fine-grained, actor-level evidence to a growing body of qualitative work on GBV in humanitarian and fragile settings [25–27], while providing specific, actionable findings grounded in the institutional and social context of Sierra Leone.

Two boundaries apply throughout. The evidence comes from 3 of the 16 districts, so relevance elsewhere is a matter of analytical transferability, and national implications are inferences from district evidence. All emergency-related evidence is retrospective, covering events 8–10 years before interview for Ebola and 1–2 years for the most recent floods, and is subject to recall bias and reconstruction; case by case elicitation, independent accounts from both sides of each referral link, and consistency with the documentary record and published analyses of the Ebola and COVID-19 periods [7,8,28] increase confidence in the broad pattern but do not substitute for observation during an active emergency.

### Clinical readiness and the emergency contraception gap

The EC and PEP findings extend evidence from comparable settings. A systematic review of sexual and reproductive health services during disaster response found EC stockouts common during emergency periods [29]; this study adds provider refusal on personal moral grounds as a distinct barrier operating independently of supply, consistent with evidence that conscientious objection without an institutional override policy denies survivors their entitlements [30]. Late presentation after the 72-hour window reflects distance, transport costs, lack of awareness, and time spent navigating informal community responses rather than survivor reluctance [31]. The redeployment of trained staff recalled in all three districts contradicts the Health EDRM requirement to maintain essential services, including GBV care, during emergencies [12]; on participants’ accounts, planning included no GBV staffing protocols, minimum standards, or surge arrangements, so the emergencies that increase risk also reduce the capacity to respond [28,32].

### Confidentiality, stigma, and the social ecology of silence

The confidentiality failures operate at several levels at once [14]. Absent private space reflects infrastructure deficits well documented in Sierra Leone but rarely examined for GBV care [33]; questioning survivors in public is a recognised form of mistreatment that training alone cannot correct without structural change [34]; and norms that construct GBV as a family matter and equate disclosure with shame deter help-seeking, the more so when displacement increases surveillance. Community leaders’ simultaneous concern for survivors and endorsement of informal settlement matches evidence from other West African settings [35] and from customary justice in Sierra Leone, where survivor interests are subordinated to family honour [36]; it reflects rational calculation where formal justice is inaccessible or retraumatising, and bears hardest on adolescent and young women [37]. The intensification of stigma in shelters is consistent with humanitarian evidence [4,38,39], and because displaced survivors withheld reports for fear of community exposure rather than of perpetrators, safe reporting channels must be separated from shared living space.

### Referral pathways and the coordination deficit

Formal referral protocols existed in every district, while the infrastructure to implement them was reported as absent, a gap that is widest in under-resourced settings and under system stress [40], and dependence on personal relationships means the system fails when key individuals are unavailable or transferred. The absence of GBV from emergency operations centres, which participants reported had no focal points, no tracking of GBV service disruption, and no GBV agenda item, mirrors under-prioritisation documented internationally [27]; the Inter-Agency Standing Committee (IASC) guidelines, endorsed within Sierra Leone’s humanitarian architecture, require GBV focal points from the earliest phase of response [4], and participants in these three districts did not experience these requirements as being met. Repeated disclosure to multiple actors without a shared record is a form of institutional retraumatisation documented elsewhere [26], and no single actor can identify escalating risk.

### Psychosocial care and the limits of survivor-centred practice

The limited availability of psychosocial support at peripheral facilities, and its reported disruption during previous emergencies, is the most acute gap identified. Only a minority of women experiencing violence access psychosocial services, and coverage is lower still in fragile settings [41]; concentrating counselling in one-stop centres leaves most survivors outside Freetown unserved and is not integrated into the public system in a way that would function during emergencies. Survivor-centred care requires time, privacy, skilled practitioners, and institutional cultures that prioritise autonomy over efficiency [42,43], and emergencies compress all of these; that sincere providers became paternalistic under pressure argues for training that addresses emergency scenarios and is reinforced by protocol. Survivors who went home without follow-up face a raised risk of post-traumatic stress disorder, depression, and suicidality [44], and guided self-help and task-shared interventions delivered by trained lay providers offer a plausible model for peripheral facilities and shelters [45].

The survivor evidence is partial in a knowable direction. All six survivors were recruited through organisations already supporting them, so every account belongs to a person who reached services and was retained; those who never disclosed, were turned away, withdrew, or whose cases were settled informally, together probably the majority [31,32], are absent. The bias is therefore conservative: the failures described here persist even among survivors who entered the system, and on what happens to a survivor who does not arrive the sample is uninformative, so research recruiting through community channels is needed.

The findings also identify entry points that already exist. The one-stop model was described as functioning by every actor category for transferable reasons, co-location, a single shared case record, and a dedicated commodity stock, which can be reproduced at district hospital level without a full centre. The FSU network is under-resourced but present, trusted in some districts, and already the designated referral destination, so standardising information transfer to it is a smaller intervention than building a new pathway. The informal messaging group that sustained referrals during COVID-19 argues for a low-technology, offline-capable communication protocol with official standing and named role holders rather than for a system requiring infrastructure the districts lack.

### Recommendations

Six recommendations follow from the findings and are mapped to the findings that motivate them and to lead actors in Table 3; each is labelled directly evidenced where it follows from what participants described, and evidence informed where this study establishes the problem but the solution is drawn from international guidance; full statements are in Supplementary file 5. In brief, embed GBV response in national emergency preparedness frameworks, with focal points in every district emergency operations centre (evidence informed [4,27,46]); enforce minimum clinical standards of private space, a trained responder, pre-positioned EC and PEP, and an institutional policy overriding provider refusal (directly evidenced for the standards, evidence informed for the override [11,16,30]); adopt a unified referral form and shared case management system with offline backup (directly evidenced for the problem, evidence informed for the platform [27,47,48]); extend psychosocial first aid training to community health volunteers, women’s groups, and primary facility staff, and deploy community-based support workers linked to district counselling (evidence informed [45,46]); engage community and religious leaders through structured, rights-based sensitisation, treating leaders who already require FSU referral as partners (evidence informed [49]); and deliver survivor-centred care training for emergency scenarios, with supervision and survivor feedback (directly evidenced [24]).

**Table 3.** Priority findings mapped to recommendations and lead actors.

Priority finding	Recommended action	Lead actors
GBV absent from emergency coordination	Appoint focal points and embed minimum standards in emergency preparedness plans	National disaster and health authorities; district emergency centres
EC and prophylaxis stockouts and provider refusal	Pre-position commodities; enforce an override policy on provider refusal	Ministry of Health; district health management teams
Fragmented, personal relationship dependent referral	Adopt a unified referral form and shared case management platform with offline backup	Health, police, social welfare, and NGO partners
Psychosocial support absent at periphery	Train and deploy community based psychosocial workers with district links	Ministry of Health; NGO partners
Ambivalent community leadership	Deliver structured, rights-based leader sensitisation linked to preparedness	Social welfare ministry; community structures
Paternalistic, inconsistent survivor centred care	Deliver emergency oriented survivor centred training with supervision and feedback	All response sectors

### Strengths and limitations

The study integrates two qualitative methods across nine actor categories and three contrasting districts, allowing triangulation between protocol and reported practice and between provider and survivor accounts of the same pathways; negative case analysis was applied systematically; and survivor perspectives ground the analysis in lived experience. Its limitations are these. Purposive sampling in 3 of 16 districts means the findings describe those districts, and national implications are inferences from district evidence. The six survivors were all engaged with services, a selection bias whose direction is conservative. Social desirability may have shaped provider and police responses. Data were collected outside an active emergency, so emergency findings rest on retrospective accounts subject to recall bias and reconstruction and are reported throughout as participant experiences and recollections rather than observed performance. Mapping relied on participant recall and available documents because routine records were incomplete, and judicial actors beyond the police and district health management team members were not sampled systematically. Finally, some nuance may have been lost in translation despite the quality assurance applied.

## Conclusions

In three districts of Sierra Leone, participants described a GBV response that was inadequate in stable conditions and, in their recollection of previous emergencies, substantially weakened: a hidden emergency within every emergency, falling on the women and girls most harmed by violence and least served by the systems meant to protect them. The components participants described as working, the one-stop model, the FSU network, and the informal communication arrangements that sustained referrals when formal channels failed, are the most feasible entry points for embedding survivor-centred care in emergency preparedness. Whether this pattern holds in the remaining districts, and how the response performs when observed during an emergency rather than recalled after one are questions for further research.

## Supplementary Material

Additional file 1 COREQ 32 item checklist.docx

GBV Sierra Leone SUPPLEMENTARY FILES 1 to 5.docx

## Data Availability

The datasets generated and analysed during the current study are not publicly available because they contain sensitive information that could compromise the privacy and safety of survivors and other participants. De-identified excerpts and the interview and mapping tools are available from the corresponding author on reasonable request and subject to ethical approval.
