# Supplementary material for: Gender-based violence response and health system readiness during emergencies in Sierra Leone: a qualitative study of survivor experience, facility preparedness, and referral pathways
Source: Glob Health Action. 2026 Sep 29;19(1):2740361. doi: 10.1080/16549716.2026.2740361 (PMC13629803; doi:10.1080/16549716.2026.2740361)
Supplement: Supplementary file 1 [file ZGHA_A_2740361_SM9599.docx]

*Supplementary material*

**Gender-based violence response and health-system readiness during emergencies in Sierra Leone**

*Additional files accompanying the manuscript.* Supplementary file 1. Interview guides and mapping of guiding frameworks to guide items; Supplementary file 2. Referral pathway mapping tool; Supplementary file 3. Extended methods; Supplementary file 4. Extended results and additional quotations; Supplementary file 5. Recommendations in full with evidence labels. Supplementary files 3 to 5 reproduce, without alteration of content, passages that were moved from the main text to meet the word limit of the journal.

**Supplementary file 1. Interview guides and mapping of guiding frameworks to guide items**

The core guide below was used with all key informant groups. Role-specific modules were appended to it as set out in the Methods of the main manuscript. Every item was probed at the individual, relationship, community, and structural levels in accordance with the social-ecological model, and the probes are not reproduced separately here.

## **A. Core guide: Domain 1. Clinical readiness and commodity availability**

1. Please describe, step by step, what happens when a survivor of GBV presents at your facility or office.

2. What commodities, equipment, and space does that response require, and which of them are reliably available?

3. Tell me about the availability of emergency contraception over the past 12 months. What determines whether a survivor receives it, and what happens when she does not?

4. Tell me about post-exposure prophylaxis for HIV. Within what time frame do survivors usually present, what happens when they present late, and why do they present when they do?

5. Who here is trained to conduct a clinical examination and to complete the medico-legal form? What training have they had, and when was it last refreshed?

6. What happens to that capability when the trained person is absent?

## **A. Core guide: Domain 2. Confidentiality, privacy, and provider conduct**

7. Where does a consultation with a survivor take place? Who else can see or hear it?

8. How is information about a case recorded, stored, and shared, and who has access to it?

9. What does consent mean in the care of a survivor, and how is it obtained in practice?

10. Are there circumstances in which a survivor is treated differently from other clients or patients? Please describe them.

11. What happens when confidentiality is not maintained, and what recourse does a survivor have?

## **A. Core guide: Domain 3. Referral and inter-agency coordination**

12. Take me through the last three GBV cases you personally handled. For each one: where did the survivor go next, whom did you contact, by what means, what information did you transfer, how long did each step take, and what resources did it require?

13. Which of those steps work reliably and which do not? What happens when a step fails?

14. What formal agreements, forms, or protocols govern referral between your organisation and the others involved?

15. How do you find out what happened to a survivor after you referred her?

16. Which links in that chain depend on particular individuals rather than on a system?

## **A. Core guide: Domain 4. Psychosocial support and survivor-centred practice**

17. What psychosocial support is available to a survivor at this point of care, and who provides it?

18. How are decisions made about what a survivor should do next, and what part does she play in making them?

19. Describe a case in which the survivor wanted something different from what you recommended. What happened?

20. What support do staff themselves receive for this work?

## **A. Core guide: Domain 5. Emergency specific experience and preparedness**

21. Think of a specific emergency you worked through. Describe what happened to GBV services during it.

22. Take one GBV case you handled during that emergency and trace the same steps we discussed in Domain 3.

23. What changed about staffing, supplies, space, and referral during that period, and what was restored afterwards?

24. Does GBV appear in the emergency plans, agendas, or coordination structures you are part of, and in what form?

25. If a comparable emergency began next month, what would happen to a survivor presenting on the first day?

26. What would need to be in place beforehand for that to go differently?

## **B. Role specific modules**

Healthcare providers: commodity ordering and stock management; clinical management of rape; medico-legal documentation and court requirements.

Police family support unit officers: case intake, evidence handling, charge and prosecution, and interaction with health facilities.

Social welfare officers: case follow-up, family mediation, placement and safe shelter, and caseload and transport.

NGO protection officers: programme coverage, funding cycles, case management systems, and inter-agency coordination.

Community and religious leaders: community norms, informal settlement practice, and their own understanding of their role.

Women’s group representatives and community health volunteers: first contact, community level referral, and what they are and are not equipped to do.

Psychosocial counsellors: caseload, supervision, continuity of care, and follow-up.

District emergency response coordinators: emergency operations centre structure, agenda setting, service continuity planning, and focal point arrangements.

## **C. Survivor guide**

The survivor guide was a narrative journey guide with no fixed question order. It contained no questions about the index event, the perpetrator, or any identifying circumstance, since the study concerned the service response. The opening invitation was: ‘Please tell me, in your own words and starting wherever you wish, what happened when you looked for help.’ Follow-up prompts, used only where the participant had already raised the topic, covered: the first person or place she went to and why; how she was received; whether she was able to speak privately; what she was told, given, or offered; what she was asked to do next and whether she was able to do it; who, if anyone, followed up with her; what support she received afterwards; what she would have wanted to be different; and what she would want a person in her position to be able to expect. The counsellor agreed stopping rules applied throughout, and the participant was reminded before and during the interview that she could decline any question or end the interview without explanation.

## **D. Mapping of guiding frameworks to guide items**

Survivor-centred care framework: items 7 to 11 and 17 to 20, covering consent, confidentiality, autonomy, non-discrimination, and safety.

Health Emergency and Disaster Risk Management framework: items 21 to 26, together with items 14 and 15, covering preparedness planning, essential service continuity, surge staffing, commodity pre-positioning, and coordination.

Social-ecological model: applied as the probe structure within every item rather than as a separate domain, so that each topic was explored in turn at the individual, relationship, community, and structural levels.

Rights-based approach to health: items 9, 11, 24, and 26, covering entitlement, accountability, and redress, asked of coordination, policy, and NGO respondents.

Items 1 to 6 derive from the clinical readiness components specified in the WHO clinical and policy guidelines and are informed by all four frameworks jointly.

**Supplementary file 2. Referral pathway mapping tool**

Referral pathway mapping was conducted as a structured three-step procedure, summarised in the Methods of the main manuscript and described in full in Supplementary file 3, section S3.6. The tool below structured the mapping discussions. The tool below structures that mapping; each link in the pathway is characterised on the dimensions in Table S1 and the documented-versus-actual comparison is recorded in Table S2.

## **Section 1. Pathway steps to be mapped**

- Entry / first point of contact (community health volunteer, facility, police, NGO, hotline).
- Clinical care (examination, emergency contraception, PEP, documentation / GBV medical form).
- Referral to police Family Support Unit (investigation, protection).
- Referral to social welfare (case management, family support).
- Referral to legal aid / justice.
- Referral to psychosocial / mental-health support.
- Referral to safe shelter / protection (including in displacement settings).
- Follow-up, case review and continuity of care.

## **Section 2. Characterisation of each link (Table S1)**

| **Dimension** | **Prompt** | **Response (per link)** |
| --- | --- | --- |
| Responsible actor | Who is responsible for this step, and is the role defined in a protocol? | … |
| Trigger & procedure | What triggers the referral, and what is the documented procedure? | … |
| Communication mechanism | How is the referral communicated (phone, form, in person)? Is there a backup if communications fail? | … |
| Transport & logistics | How does the survivor or information physically move? Who pays? | … |
| Timeframe | How long does this step take in practice? Is any time-critical window involved (e.g. 72 hours)? | … |
| Information shared | What information accompanies the referral? Is a shared/unified form used? | … |
| Breakdown points | Where and why does this link fail — routinely, and specifically during emergencies? | … |
| Emergency change | How does this step change during an emergency (staffing, access, coordination)? | … |

## **Section 3. Documented protocol versus functional reality (Table S2)**

| **Pathway link** | **Documented protocol (on paper)** | **Functional reality (stable period / emergency)** |
| --- | --- | --- |
| Entry / first contact | … | … |
| Clinical care (EC, PEP, documentation) | … | … |
| Health → police (FSU) | … | … |
| Health → social welfare | … | … |
| Referral to legal aid | … | … |
| Referral to psychosocial support | … | … |
| Safe shelter / protection | … | … |
| Follow-up & case review | … | … |

*EC, emergency contraception; FSU, Family Support Unit; PEP, post-exposure prophylaxis. The completed maps informed the referral-pathway figure in the main manuscript comparing the documented protocol with the functional reality during emergencies.*

**Supplementary file 3. Extended methods**

This file reproduces, without alteration of content, the methodological passages that were moved from the main text of the manuscript to meet the word limit of the journal. Section numbers are referred to from the Methods of the main manuscript, and reference numbers refer to the reference list of the main manuscript.

## **S3.1 Survivor recruitment, safeguards, and analytic role of survivor interviews**

Survivors were recruited indirectly and were never approached by the research team in the first instance. Two organisations already providing them with ongoing psychosocial support, the Rainbo Initiative and a partnering NGO, identified clients who met the eligibility criteria set out below, and the supporting counsellor, not the research team, made the initial approach and provided a written and oral study information sheet. Only those who then contacted the team, or who asked their counsellor to pass on their agreement to be contacted, were enrolled. No screening tool or symptom scale was applied; the judgement of clinical stability was made by the supporting counsellor, who knew the client, against three written criteria agreed with the organisations in advance: that at least six months had elapsed since the index event, that the client was not in acute crisis or ongoing danger from a perpetrator, and that the counsellor judged participation unlikely to interfere with the client’s ongoing support. In this study, rigorous ethical review therefore denotes a defined package of measures rather than a general assurance: review and approval of the survivor protocol as a separate component by the Sierra Leone Ethics and Scientific Review Committee (SLESRC); indirect recruitment through a supporting counsellor; interviewing only by female researchers with GBV counselling backgrounds; a written distress protocol with defined stopping rules; a named counsellor available on the day of each interview; the participant’s right to end the interview or omit any question without explanation; a location chosen by the participant; and no collection of identifying detail about the index event or the perpetrator, since the study concerned the service response and not the violence itself.

In the analysis, survivor transcripts were coded within the same framework as key informant transcripts and were not treated as a separate dataset, so that survivor and provider accounts of the same points in the care pathway could be read against each other in the framework matrix. Survivor accounts served three specific analytic functions. They were used to test provider accounts of what happens at the point of care, and the divergences that emerged are reported explicitly in the Results. They were used to validate the referral pathway maps derived from provider accounts, by comparing the route a survivor described taking with the route providers described offering. They were not used to estimate the frequency or distribution of any experience, and no claim of saturation is made for the survivor group.

## **S3.2 Eligibility criteria**

Key informants were eligible if they were adults aged 18 years or older; had held a role carrying defined GBV response, service delivery, or coordination responsibilities in one of the study districts for a minimum of two years; had been personally involved in the management, referral, or coordination of at least one GBV case in the 12 months preceding the interview; and provided voluntary informed consent. Survivors were eligible if they were adults aged 18 years or older, had experienced GBV at least six months before the interview, were already receiving psychosocial support from a referral organisation, and were assessed by their supporting counsellor as clinically stable for participation. Individuals in acute crisis, in ongoing danger from a perpetrator, or in active clinical care for the index event were not approached.

## **S3.3 Assessment of thematic saturation**

Recruitment continued until thematic saturation was reached. Saturation was assessed prospectively and separately for each stakeholder group rather than for the sample as a whole, using the code-based approach described by Hennink and Kaiser [22]. Transcripts were coded in batches of four to six, and after each batch the two coders recorded the number of new codes generated and whether any new code altered an existing subtheme. Saturation for a stakeholder group was defined a priori as two consecutive batches yielding no new codes and no modification to the analytical framework for that group. On this definition, saturation was reached after 9 of the 11 healthcare provider interviews, after 5 of the 6 social welfare interviews, after 5 of the 6 FSU interviews, after 6 of the 7 NGO interviews, and after 4 of the 5 interviews in each of the remaining key informant groups; the additional interviews in each group were nonetheless completed and confirmed that no new codes emerged. For survivors, the small number of interviews means that saturation was neither sought nor claimed, and these interviews are treated as illustrative of experience rather than as an exhaustive account. Saturation decisions were recorded in a saturation log maintained in NVivo alongside the codebook, were reviewed at three scheduled analysis meetings, and were agreed by all authors before recruitment closed.

## **S3.4 Operationalisation of the guiding frameworks and role-specific modules**

The four guiding frameworks were operationalised as distinct question domains rather than being invoked in general terms. The survivor-centred care framework [24] generated the items on consent, confidentiality, autonomy, non-discrimination, and safety, which constitute domains 2 and 4. The Health EDRM framework [12] generated the items on preparedness planning, essential service continuity, surge staffing, commodity pre-positioning, and coordination structures, which constitute domain 5 and part of domain 3. The social-ecological model [14] was not used to generate a separate domain but to structure the probes within every domain, so that each topic was explored in turn at the individual, relationship, community, and structural levels. The rights-based approach to health [15] generated the items on entitlement, accountability, and redress, which were asked of coordination, policy, and NGO respondents within domains 3 and 5. All four frameworks therefore informed the guides, and the item-level mapping is set out in Supplementary file 1.

A single core guide was used with all key informant groups, in order to preserve comparability across the five domains, and role-specific modules were appended to it. Healthcare providers received additional items on commodity stock and ordering, clinical management of rape, and medico-legal documentation; FSU officers on case handling, evidence, and prosecution; social welfare officers on case follow-up, family mediation, and placement; NGO officers on programme coverage, funding, and coordination; community and religious leaders on norms, informal settlement, and their own role expectations; women’s group representatives and community health volunteers (CHVs) on first contact and community level referral; psychosocial counsellors on caseload, supervision, and continuity; and district emergency response coordinators on emergency operations centre structures, agenda setting, and service continuity planning. The survivor guide was constructed separately as a narrative journey guide that followed the participant’s own account of the sequence of events and services, with no fixed question order, no questions about the index event itself, and the counsellor agreed stopping rules applied throughout.

## **S3.5 Transcription, translation, and quality assurance**

Interviews were audio recorded and transcribed in the language of the interview by two bilingual transcribers who were members of the research team and had received project-specific training in verbatim transcription and in GBV research ethics. Krio and Mende transcripts were translated into English by the transcriber who had produced them. A second bilingual team member then reviewed every translated transcript against the original recording, checking for omission, for added meaning, and for terminology. A randomly selected 20% of translated transcripts was additionally back-translated into the source language by a third bilingual researcher who had not been involved in that interview, and the back-translation was compared with the original transcript. Discrepancies were logged and resolved in a translation review meeting; of the 34 discrepancies logged, 29 concerned terminology and 5 concerned emphasis, and none altered the code assigned to a segment. Culturally specific terms with no direct English equivalent, including the Krio expressions used for shame, for settling a matter, and for the family meeting through which such matters are resolved, were retained in the source language in the transcript with a glossed English rendering in square brackets, and were coded on the source term so that meaning was not lost at the coding stage. Coding was carried out on the reviewed English transcripts with the source language transcript available alongside, and every quotation reported in this paper was re-checked against the source recording before inclusion.

## **S3.6 Referral pathway mapping procedure**

Referral pathway mapping was conducted as a structured three-step procedure. First, a documented pathway was reconstructed for each district from written sources: the 2018 national GBV referral protocol, district GBV coordination minutes where these were available, facility standard operating procedures, FSU case handling instructions, and the referral forms in use at each site. Second, a functional pathway was elicited from participants. In 22 mapping discussions, 9 in Western Area Urban, 7 in Kenema, and 6 in Port Loko, healthcare providers, social welfare officers, FSU officers, and NGO protection officers were asked to trace, step by step, the route taken by the last three GBV cases they had personally handled. For each step participants specified the receiving actor, the mode of contact, the information transferred, the time elapsed, the resources required, and what happened when that step failed. Third, each participant was asked to repeat the exercise for a case handled during a previous emergency, so that the same steps could be compared across stable and emergency conditions.

Each discussion produced a hand-drawn node and link diagram in which nodes represented actors or service points and links represented referral steps. Links were coded on three attributes: whether the step was specified in protocol, whether it was reported as routinely completed in practice, and what the reported point of failure was. Diagrams were transcribed into a common notation by two researchers working independently and were reconciled by discussion, then compiled into a district-level matrix in which rows were referral steps and columns were the three attributes, recorded separately for stable and for emergency conditions. The three district matrices were then compared to produce the cross-district synthesis presented in Figure 4. A link is shown as functional in Figure 4 only where it was reported as routinely completed by participants from both the referring and the receiving actor group; where accounts diverged, the divergence was retained and is reported in the Results rather than resolved in favour of either account.

Three validation steps were applied. First, participant-reported steps were cross-checked against the documentary sources and against the accounts of the receiving actor, so that no link in Figure 4 rests on a single respondent group. Second, draft district maps were taken back to 11 participants across the three districts, at least three per district and drawn from different actor categories, who were asked to correct, add, or remove steps; the corrections received concerned the sequence of social welfare notification in Port Loko and the role of the district health management team in Kenema, and both were incorporated. Third, survivor accounts of their own journeys were compared with the provider-derived maps, and the points at which survivor accounts diverged from provider accounts are reported explicitly in the Results. The mapping was not audited against routine service records, which were incomplete or unavailable at most sites, and this is acknowledged as a limitation. Because the emergency condition maps were elicited retrospectively, they represent participants’ reconstruction of past referral practice and not observed performance during an emergency.

## **S3.7 Application of the framework method**

The framework method was applied in the following way [18,19]. In stage one, five transcripts spanning different stakeholder groups and districts were read repeatedly by two researchers, who recorded impressions in familiarisation memos. In stage two, those five transcripts were open-coded line by line, generating 214 initial codes, which were grouped into a working analytical framework of 4 categories and 14 subcategories; the framework combined inductive codes arising from the data with deductive codes derived a priori from the four guiding frameworks, and every code was given a written definition together with inclusion and exclusion rules in a codebook. In stage three, the working framework was applied to all 54 transcripts, with the codebook formally revised twice during indexing as new codes were added; the final framework comprised 4 themes, 11 subthemes, and 96 codes. In stage four, indexed data were charted into a framework matrix in NVivo with one row per participant and one column per subtheme, so that each cell held the summarised and referenced data for that participant on that subtheme. In stage five, the matrix was interrogated by column to characterise each subtheme across the sample, and by row to preserve the coherence of individual accounts, and was read across stakeholder groups and districts to identify convergence, divergence, and deviant cases. Double coding was applied to 20% of transcripts. Intercoder agreement was assessed through structured discussion rather than through a numerical coefficient, consistent with the interpretive assumptions of the method; the 17 disagreements recorded were resolved by consensus with reference to the codebook, which was amended in 4 instances as a result.

## **S3.8 Trustworthiness, negative case analysis, and reflexivity**

Trustworthiness was addressed against the four criteria of credibility, transferability, dependability, and confirmability [18]. Credibility was strengthened through method triangulation across interviews and referral pathway mapping, source triangulation across nine stakeholder categories and survivors, and the cross-referencing of provider and survivor accounts of the same care pathways. Transferability was supported by purposive selection of three districts spanning urban, mixed, and rural conditions and by thick description of the study context. Dependability was supported by independent double coding, consensus resolution, and an audit trail of coding decisions maintained in NVivo. Confirmability was supported by reflexive memos and by negative case analysis. Negative case analysis was conducted systematically rather than incidentally: at the end of stage five, each subtheme was deliberately re-interrogated for accounts that qualified or contradicted it, and every such account was extracted into a separate deviant case file. The contents of that file are reported in full in a dedicated Results subsection of the main manuscript and in full in Supplementary file 4 rather than being absorbed into the dominant themes. The research team included Sierra Leonean and international researchers with backgrounds in GBV programming, emergency health, public health, and clinical medicine. Team members reflected on their own positionality throughout the research process, acknowledging that institutional affiliations and professional experience may shape interpretive perspectives.

**Supplementary file 4. Extended results and additional quotations**

Section S4.1 provides additional illustrative quotations for each theme, with the framing text that accompanied them in the previous version of the manuscript, and section S4.2 reproduces in full the subsection reporting divergent accounts, deviant cases, and components that functioned. Participants are identified by role and district only. Emergency-related accounts are retrospective and are reported as participant experiences and recollections rather than as directly observed system performance.

## **S4.1 Additional illustrative quotations by theme**

## **Theme 1: Fragmented and under-resourced clinical readiness**

In Freetown, a medical officer at a referral hospital noted that even where EC was physically available, provider attitudes created barriers to access:

*Some of my colleagues refuse to give emergency contraception. They say it is against their beliefs. There is no clear policy that overrides this. The woman comes in, she has been raped, and she is sent away or told to come back tomorrow. Tomorrow is too late. (Medical officer, Western Area Urban)*

PEP for HIV was similarly affected. Healthcare providers in Kenema described a 72-hour window that was routinely missed because survivors arrived late, were turned away from the first facility they attended, or were not informed that PEP was available:

*Most of our survivors come after 72 hours. By then, post-exposure prophylaxis is not effective. They did not know they had to come quickly. Nobody told them. The community does not know this. (Nurse, Kenema)*

The capacity to conduct forensic clinical examinations was severely limited outside Freetown. In Port Loko and Kenema, only one or two clinicians per district had received any training in clinical management of rape, and in both districts those individuals had received no refresher training in more than two years. Medical documentation, including the completion of GBV medical forms required for legal proceedings, was described as inconsistent and poorly understood:

*I was never trained to fill the gender-based violence form properly. I fill what I can but I know it is not complete. When the police come and ask for the form, I am embarrassed. The form is supposed to help the survivor in court but if it is wrong it helps no one. (Medical officer, Kenema)*

Participants recalled that during previous emergencies the documentation problem was compounded by staff redeployment. In all three districts, participants described scenarios in which the one or two clinicians with GBV training were assigned to emergency response duties, leaving facilities without any trained responder:

*During Ebola, during floods, the trained people go to the emergency. The facility is left with whoever is available. Those people have no training. They do not know what to do with a gender-based violence case. They send the woman away or they call someone on the phone who is busy. (District emergency coordinator, Western Area Urban)*

## **Theme 2: Confidentiality failures and stigma as structural barriers**

Healthcare providers described the same conditions from their own vantage point, locating the difficulty in the physical infrastructure available to them:

*We do not have a private room. We have one consultation room and it is always full. I try to speak quietly but the walls are thin. I know the woman is uncomfortable. I wish I could do better but I have nothing to work with. (Nurse, Port Loko)*

Women's group representatives offered a sharply contrasting perspective, describing the pressure to settle as a mechanism that protected perpetrators and silenced survivors:

*The community says settle, settle, settle. The chief calls a meeting, the man pays a fine, and the woman is told to forgive and move on. She has no voice in that meeting. Nobody asks what she wants. Nobody asks if she is safe. (Women's group representative, Kenema)*

During emergencies, stigma was intensified by displacement and community disruption. Survivors who had been displaced from their home communities into temporary shelters described heightened surveillance, loss of social networks, and increased exposure to perpetrators in confined spaces:

*In the shelter, everyone knows everyone's business. There is no privacy. If something happens to you, the whole camp knows by morning. So, women do not report. They are afraid of what people will say. (Survivor, Western Area Urban)*

## **Theme 3: Dysfunctional referral pathways and inter-agency coordination gaps**

Referrals from health facilities to police FSUs were described as inconsistent, delayed, and frequently dependent on the personal initiative of individual staff members rather than systematic protocols. Police family support personnel expressed frustration with receiving incomplete referrals and at the absence of standardised information sharing procedures:

*Sometimes the hospital calls us, sometimes they do not. When they call, they give us very little information. We arrive, and the survivor has already left. Or the clinician who saw her has gone off duty, and nobody else knows what happened. There is no system. It is all personal relationships. (Family Support Unit officer, Western Area Urban)*

Social welfare officers described similar fragmentation, compounded by their own resource constraints:

*I cover three chiefdoms. I have no vehicle, no phone credit, and no budget for transport. When a referral comes, I try to respond, but sometimes I cannot reach the family for days. By then the situation has changed. The woman may have been pressured to withdraw. (Social welfare officer, Port Loko)*

Community health volunteers, who often served as the first point of contact for survivors in rural areas, reported receiving no training in GBV referral and no clear guidance on where to direct survivors:

*I know something bad has happened to a woman in my community. I want to help her. But I do not know who to call. I do not know the number for the Family Support Unit. I do not know if the clinic will help her or turn her away. I am trying to help, but I am doing it in the dark. (Community health volunteer, Port Loko)*

NGO protection officers described a coordination landscape that was fragmented in stable periods and dysfunctional during emergencies. Information sharing between organisations, government health facilities, and police was inconsistent, and there was no unified case management system across actors:

*We all work in the same district, but we do not share information. Each organisation has its own database, its own referral form, its own process. The survivor has to tell her story three times, four times, to different people. Every time she tells it, she is traumatised again. (Non-governmental protection officer, Western Area Urban)*

## **Theme 4: Limited and inconsistent survivor-centred psychosocial care**

In Freetown, psychosocial counsellors were available at Rainbo Initiative one-stop centres but were absent from the majority of peripheral health facilities. In Kenema and Port Loko, counsellors were present in district hospitals but not in community health centres or health posts. Participants recalled that during previous emergencies even these limited counselling resources were disrupted:

*When the emergency happens, the counsellors are sometimes redeployed, or they cannot reach the facility. The survivors who need support the most are left with nothing. A nurse gives them a tablet and sends them home. (Psychosocial counsellor, Kenema)*

The survivor-centred care framework, while referenced in national GBV protocols, was poorly understood and inconsistently applied at facility level. Several healthcare providers described directing survivors towards particular courses of action:

*We try to help the woman, but sometimes we know what is best for her. We tell her to report to the police, to take the medicine, to go to the shelter. She may not want all of these things, but we push because we want to help. (Medical officer, Western Area Urban)*

NGO protection officers were more consistently articulate about survivor-centred principles, but acknowledged that emergency conditions created pressure to prioritise efficiency over agency:

*In an emergency, everything is fast. You want to move the survivor to safety, get her medicine, get her a referral. But you forget to ask her what she wants. You forget that she is the one who has to live with the decisions. Survivor-centred care takes time and emergencies do not give you time. (Non-governmental protection officer, Kenema)*

## **S4.2 Divergent accounts, deviant cases, and components that functioned (full text)**

Negative case analysis identified accounts that qualified or contradicted each of the four themes. They are reported here in full rather than absorbed into the dominant narrative, because they bear directly on which components of the existing response could be strengthened.

The clearest counter case concerned the Rainbo Initiative one-stop centres. Healthcare providers, FSU officers, NGO staff, and survivors in Western Area Urban and Kenema described the one-stop model as the part of the system that worked as intended, and their accounts were consistent with one another and with the documented protocol. Participants attributed this to three specific features: the co-location of clinical care, counselling, and a police desk within a single confidential building; a standing case file shared across those three functions, so that the account did not have to be repeated; and a dedicated commodity stock held by the centre rather than drawn from the general facility pharmacy. Two of the six survivors had reached a one-stop centre, and both described an experience that differed materially from the accounts of those who presented at a general facility.

*At that place they took me into a room and closed the door. One person asked me the questions, not everybody. The medicine was there that same day. The police person came to me, I did not have to go to the station. I told my story one time. (Survivor, Western Area Urban)*

Participants also identified functioning practice outside the one-stop model. In Western Area Urban, FSU officers and hospital staff independently described an informal messaging group linking the FSU duty officer, two hospital clinicians, and an NGO caseworker, which participants reported had sustained referrals through the communication disruptions of the COVID-19 period, when formal channels were suspended. Participants described this arrangement both as effective and as fragile, because it rested on the particular individuals who happened to be in it.

*The group works. If I put a case there, somebody answers, even at night. But it is not written anywhere. If those people transfer tomorrow, the group is finished, and nobody will know that it was the group holding everything together. (Family Support Unit officer, Western Area Urban)*

Accounts of community leaders were similarly divided. Alongside the accounts of informal settlement reported under Theme 2, participants in Kenema and Port Loko described section chiefs who required that suspected cases be taken to the FSU, and one NGO officer described a chiefdom bye-law making the concealment of a sexual offence a sanctionable act. Women’s group representatives who were critical of community leadership in general nevertheless distinguished between individual leaders on this point, rather than describing leaders as uniformly obstructive.

Two further divergences qualify the clinical findings. First, as noted above, provider refusal to supply EC was reported in Western Area Urban and Kenema but was not described by any participant in Port Loko, so the two barriers to EC access are not uniformly distributed and would not be addressed by a single intervention. Second, three healthcare providers reported keeping a small personal reserve of EC and PEP outside the main store for use when the facility was out of stock. Participants described this unofficial practice in two ways at once: as the reason that some survivors received timely treatment, and as evidence that the formal supply system was not functioning.

Survivor accounts were not uniformly negative. Of the six survivors interviewed, two described the clinical care they received as respectful and adequate, and four identified at least one individual provider or counsellor whom they described as having helped them. Where survivors were critical, the criticism was directed more often at the organisation of services, at the absence of privacy, and at what happened after the clinical encounter, than at the individuals who attended to them. This distinction between individual conduct and system design recurs across the survivor accounts and is taken up in the Discussion of the main manuscript.

**Supplementary file 5. Recommendations in full with evidence labels**

The findings generate a set of evidence-based recommendations directed at national government, district health management teams, emergency response coordinators, NGO partners, and community actors. These are summarised, with the findings that motivate them and the lead actors responsible, in Table 3 of the main manuscript. The evidentiary basis of each recommendation is stated explicitly. A recommendation is labelled directly evidenced where it follows from what participants in this study described, and evidence informed where this study establishes the problem but the specific solution proposed is drawn from international guidance and the policy literature rather than from these data. This labelling is intended to make clear where the findings of the study end and where established external frameworks are being applied to them. Reference numbers refer to the reference list of the main manuscript.

1. Embed GBV response in national emergency preparedness frameworks. National disaster management and health authorities should revise emergency preparedness plans to include minimum service standards, staffing protocols, supply pre-positioning, and coordination mechanisms, drawing on established inter-agency guidance [4,46]. Focal points should be appointed to all district emergency operations centres with defined roles, resources, and reporting lines. (Evidence informed: participants established the absence of GBV from emergency coordination in the study districts, while the specific reform proposed is drawn from inter-agency guidance and the coordination literature [4,27,46].)

2. Establish and enforce minimum clinical standards. Every facility at community health centre level and above should maintain a private consultation space, a trained clinical responder, and a minimum stock of EC and PEP, designated as essential emergency commodities with pre-positioned district reserves [11,16]. A clear institutional policy overriding individual provider refusal to provide EC should be developed and enforced, and should be accompanied by a documented duty to refer without delay. (Directly evidenced for the commodity, private space, and trained responder components, which participants identified specifically and in the same terms across districts; evidence informed for the design of an override policy, for which this study establishes the need but not the mechanism [30].)

3. Develop a unified referral and case management system. A single referral form and shared case management platform should be adopted across health, police, social welfare, and NGO actors, designed to function during communication disruptions through offline paper-based backup, and linked to child protection systems in emergencies [47,48]. Regular inter-agency case review meetings should be formalised and resourced. (Directly evidenced for the problem of repeated disclosure and fragmented records, which both providers and survivors described; evidence informed for the design of a shared platform, which is drawn from the coordination and case management literature [27,47,48].)

4. Invest in community-based psychosocial capacity. Psychosocial first aid training should be extended to community health volunteers, women's group representatives, and primary facility staff, and a cadre of community-based support workers should be recruited, trained, and deployed to peripheral facilities and shelter settings with defined links to district counselling services [45]. (Evidence informed: this study establishes the absence of counsellors at peripheral facilities, while the task-shared delivery model proposed is drawn from trial evidence in comparable settings [45] and from mental health and psychosocial support (MHPSS) guidance for emergencies [46].)

5. Implement structured community leader engagement. A programme of structured sensitisation for community and religious leaders should be developed, grounded in rights-based and violence prevention evidence, that addresses the harms of informal settlement, the rights of survivors to formal services, and the role of leaders as facilitators rather than gatekeepers of response [49]. Leaders who already require referral to the police family support unit, identified through negative case analysis in this study, should be engaged as programme partners rather than treated only as targets of sensitisation. (Evidence-informed: participants described both obstructive and facilitative leadership, while the content of the proposed programme is drawn from the prevention literature [49].)

6. Strengthen survivor-centred care training and accountability. All health, police, and social welfare personnel with response roles should receive survivor-centred care training that explicitly addresses emergency scenarios, accompanied by supervision, mentorship, and survivor feedback systems [24]. (Directly evidenced: providers themselves described the tension between survivor autonomy and the operational pressures of emergency response, and identified the training and supervision gaps that this recommendation addresses.)
