# Supplementary material for: Gender-based violence response and health system readiness during emergencies in Sierra Leone: a qualitative study of survivor experience, facility preparedness, and referral pathways
Source: Glob Health Action. 2026 Sep 29;19(1):2740361. doi: 10.1080/16549716.2026.2740361 (PMC13629803; doi:10.1080/16549716.2026.2740361)
Supplement: Supplementary file 2 [file ZGHA_A_2740361_SM9600.docx]

**Additional file 1. COREQ 32-item checklist**

Consolidated criteria for reporting qualitative research (COREQ). For each item, the table indicates how it was addressed and where it is reported in the manuscript.

| **#** | **Item** | **How addressed / location in manuscript** |
| --- | --- | --- |
| **Domain 1: Research team and reflexivity** | | |
| 1 | Interviewer/facilitator | Interviews conducted by trained researchers; survivor interviews by trained female researchers with GBV-counselling backgrounds; bilingual research assistants provided interpretation (Data collection procedures; Ethical safeguards). |
| 2 | Credentials | Team of Sierra Leonean and international researchers in GBV programming, emergency health, public health and clinical medicine (Trustworthiness and reflexivity). |
| 3 | Occupation | Academic and applied researchers and trained field research assistants (Trustworthiness and reflexivity). |
| 4 | Gender | Mixed-gender team; female researchers conducted all survivor interviews (Ethical safeguards). |
| 5 | Experience and training | Researchers experienced in GBV and qualitative methods; assistants trained in GBV-sensitive, safety-first procedures (Data collection; Ethical safeguards). |
| 6 | Relationship established | Key informants approached through district structures; survivors only through referral organisations providing ongoing support (Participants; Ethical safeguards). |
| 7 | Participant knowledge of the interviewer | Participants informed of the team, purpose and voluntary nature before consent (Core guide, Part A). |
| 8 | Interviewer characteristics and reflexivity | Reflexive memos and positionality reflection maintained throughout (Trustworthiness and reflexivity). |
| **Domain 2: Study design** | | |
| 9 | Methodological orientation | Qualitative design integrating semi-structured interviews with referral pathway mapping; thematic framework analysis (Study design; Data analysis). |
| 10 | Sampling | Purposive sampling across eight stakeholder groups plus survivors (Participants and sampling strategy). |
| 11 | Method of approach | Sampling frame built with district health management teams, police FSUs and NGO coordinators; survivors via referral organisations (Participants; Ethical safeguards). |
| 12 | Sample size | 54 participants (48 key informants + 6 survivors) (Participants and sampling strategy; Table 1). |
| 13 | Non-participation | Recruitment continued to thematic saturation; refusals not a limiting factor (Participants and sampling strategy). |
| 14 | Setting of data collection | All interviews in private settings to protect confidentiality (Ethical safeguards). |
| 15 | Presence of non-participants | Interviews conducted privately; no non-participants present (Ethical safeguards). |
| 16 | Description of sample | Participant characteristics reported in Table 1 and the study profile figure. |
| 17 | Interview guide | Core, stakeholder-specific and survivor guides, piloted with four participants, provided in Additional file 1. |
| 18 | Repeat interviews | No repeat interviews conducted (Data collection procedures). |
| 19 | Audio/visual recording | All interviews audio-recorded with consent (Data collection procedures). |
| 20 | Field notes | Analytical and reflexive memos maintained (Data analysis; Trustworthiness). |
| 21 | Duration | Interviews lasted 30–60 minutes (Data collection procedures). |
| 22 | Data saturation | Recruitment continued until no new codes or themes emerged (Participants and sampling strategy). |
| 23 | Transcripts returned | Transcripts not returned to participants; provider and survivor accounts of the same pathways were cross-referenced (Trustworthiness). |
| **Domain 3: Analysis and findings** | | |
| 24 | Number of data coders | Initial coding conducted independently by two researchers; discrepancies resolved by consensus (Data analysis). |
| 25 | Description of the coding tree | Framework identified through inductive and deductive coding, indexed and charted into a matrix (Data analysis; thematic-framework figure). |
| 26 | Derivation of themes | Themes derived through thematic framework analysis (Data analysis). |
| 27 | Software | NVivo 14 used to manage and organise coded data (Data analysis). |
| 28 | Participant checking | Not conducted with individual participants; triangulation and cross-referencing used instead (Trustworthiness). |
| 29 | Quotations presented | Verbatim quotations attributed by role and district only, to protect anonymity (Results). |
| 30 | Data and findings consistent | Findings triangulated across methods, nine stakeholder categories and survivors (Trustworthiness; Table 2). |
| 31 | Clarity of major themes | Four themes presented with subthemes (Results; thematic-framework figure). |
| 32 | Clarity of minor themes | Subthemes and divergences (e.g. provider vs survivor accounts) described (Results). |

*FSU, Family Support Unit; GBV, gender-based violence.*
